# Prevalence of early childhood caries in South Africa: a systematic review

**DOI:** 10.1186/s12903-021-01982-6

**Published:** 2022-02-08

**Authors:** Faheema Kimmie-Dhansay, Robert Barrie, Sudeshni Naidoo, Tina Roberts

**Affiliations:** 1 Division of Research and Postgraduate Studies, Faculty of Dentistry, Tygerberg Hospital - UWC Dental School, University of the Western Cape, Francie Van Zijl Drive, Parow, Cape Town, 7500 South Africa; 2 Department of Community Oral Health, Faculty of Dentistry, University of the Western Cape, Cape Town, South Africa; 3 Department of Oral Biology and Dental Genetics, Faculty of Dentistry, University of the Western Cape, Cape Town, South Africa

**Keywords:** Early childhood caries, Prevalence, South Africa, ECC

## Abstract

**Background:**

The prevalence of Early Childhood Caries (ECC) in South Africa was last determined in 2002 in a national survey. Since then only few scattered studies were conducted across pocketed communities in the country. There appears to be an increasing trend in dental caries prevalence in South Africa. Since South Africa has one of the highest burden of HIV in the world, less focus was spent on Non-Communicable Diseases (NCDs), it was only when HIV patients developed NCD’s did the government start evaluating NCDs. However, oral health is still not given enough attention within the health sphere. It is the hope of this systematic review to showcase the extent of dental caries amongst the future leaders of our country.

**Objectives:**

The systematic review presents the prevalence and severity of early childhood caries between age groups and provinces in South Africa from 1975 to 2014.

**Design:**

A systematic review of prevalence was performed. Settings and participants: All studies performed on children under the age of six who lived in South Africa were eligible to be included.

**Primary and Secondary outcome measures:**

The prevalence of ECC and the dmft scores were the primary and secondary outcome measures.

**Results:**

Twenty-one studies were included in the present review. The overall prevalence was 44.94% (95% confidence interval (C.I.) 39.73–50.15%) and the overall dmft score was 2.422 (95% C.I. 2.148–2.696).

**Conclusions:**

The dmft score was the lowest in the Limpopo Province and highest in the Eastern Cape. ECC prevalence increased post-apartheid possibly on account of lifestyle changes due to the exodus from rural to urban and peri-urban areas.

*Registration *The protocol of this systematic review was registered with PROSPERO, CRD42018112161, in November 2018.

**Supplementary Information:**

The online version contains supplementary material available at 10.1186/s12903-021-01982-6.

## Introduction

Dental caries is the progressive damage to the enamel caused by commensal bacteria in the mouth. External pathogens have not been shown to cause dental caries. However, a change in the homeostasis between normal commensals in the mouth and the surrounding tissues and structures has been shown to lead to dental caries causation [1]. Bacterial concentrations increase when there is inadequate removal of plaque and increased dietary sugar [2]. If untreated, dental caries can lead to pain and early loss of teeth, resulting in disfigurement and affect the oral health-related quality of life [3]. Early childhood caries (ECC) is defined as the presence of one or more cavitated or non-cavitated, decayed, missing (due to caries), or filled tooth in any primary (deciduous) tooth in a child aged under six years [4].

In 2017, the incidence of oral health conditions was ranked third-highest among all health problems and consisted of 3,6 billion cases, and approximately 530 million children suffer from deciduous caries globally [5]. The prevalence of ECC varies both across countries [6–8] and within the same country [7]. Similarly, the prevalence of ECC in South Africa (SA) differs between provinces. In 2004, the national prevalence of the disorder was 60.3% for 6 year olds [9]. No current national prevalence data for ECC in South Africa is available. However, Smit et al. [10] documented a significantly higher prevalence (84%) in the Western Cape than described by van Wyk et al. in 2004 [9].

The severity of the dental disease is expressed as the decayed, missing and filled tooth score and measured as the decayed, missing and filled tooth (dmft) index. International figures for the dmft indices vary: in Qatar it was reported as 7.6 [11], 3.65 in China [12], and 2.46 in Palestine [13]. In South Africa, the national dmft score was 2.4 [9], and 6.2 in the Western Cape [10].

South Africa is a densely populated developing country. It is listed as an upper- to middle-income country with 59.62 million inhabitants, of which children under the age of five constitute almost 10% or 5.7 million [14]. Historically, South Africa was immersed in political and racial division. Since the freedom charter was introduced in 2004, every South African is considered equal. Globally, South Africa has the highest income disparity within its constituents, with a Gini index of 63.0 in 2014 [15]. The Gini index determines the measure of inequality within a country. An index of 100 represents perfect inequality, and a measure of 0 means that the population is equal (all individuals have the same income) [16]. The country’s economic inequalities have resulted in an association between the prevalence of dental caries and unmet treatment needs [17].

Early Childhood Caries (ECC) has a significant burden in South Africa, particularly in the Western Cape Province. A few published studies report ECC’s prevalence in children under six years of age and under living in South Africa. To effectively prevent and manage ECC in South Africa, it is essential to know the disease prevalence and severity within this population. Therefore, the present study aimed to determine the prevalence and severity of ECC in South Africa.

## Materials and methods

This study was conducted according to the Meta-Analysis of Observational Studies in Epidemiology (MOOSE) guidelines [18], Additional file 1: Table S1. The protocol of this systematic review was registered with PROSPERO, CRD42018112161, in November 2018. The protocol paper was published in JMIR Research Protocols in 2021 [19]. Ethics approval was not required as the present investigation was not a primary study involving participants.

A comprehensive search strategy was first developed by a research team comprising experts in paediatric dentistry, epidemiology, biostatistics, and librarian studies. There was no limit to the language of publication. Although all the studies were performed in South Africa, the studies were all published in English. The first and last authors (FKD and TR) independently conducted a pilot study to test the strategy, following which the authors confirmed the final search strategy. Peer-reviewed articles were searched in the following databases until the end of November 2020, MEDLINE; ERIC via EBSCOhost; Scopus; CINAHL via EBSCO (1900 to present); Dentistry and Oral Sciences Sources via EBSCOhost; Academic Search Complete via EBSCOhost; E-Journals via EBSCOhost; Health Source: Nursing Academic Edition via EBSCOhost and Cochrane Library. Using the key terms: (a) "early childhood caries" OR "caries" OR "decay" OR "dmft" OR "dental" OR "oral" OR "PUFA" (b) "prevalence" and (c) "children" OR "peri-natal" OR "paediatric" OR "pediatric" OR "neonatal" OR "infant" and (d) "South Africa". The keywords were used in the following combinations: a + b + c + d. Hand searching of included articles was performed. All eligible studies downloaded from the databases were uploaded into Rayyan [20], where duplicates were removed.

### Screening and selection criteria

Studies were included if they were conducted in South Africa; they were based on children six years and under from the general population, if they reported sufficient information on the prevalence of ECC (sample size, prevalence of disease, mean of dmft, standard deviation of dmft) (Additional file 2: Table S2). Articles were excluded if they were abstracts, commentaries, review articles, or intervention studies. Dissertations, conference proceedings, commentaries/letters and other grey literature were also excluded from this review. Cross-sectional and cohort studies were eligible for inclusion. The inclusion and exclusion of articles were performed in Rayyan [21]. Any disagreements in the screening of articles were clarified with all the authors (Additional file 2: Table S2).

### Data extraction

Two authors independently screened (FKD and TR) and extracted data from the included articles into Excel. If there was any disagreement between the authors, a consensus was reached through discussion with all the authors. If possible and required, the corresponding authors were contacted to provide the additional or missing information. In instances where articles failed to reflect the data collection date and were too old, or the authors could not be contacted, a consensus was reached among the present study’s authors to impute a suitable missing year of data collection; usually, 3–5 years before the study was published.

The following information was extracted from each eligible study: author, year of publication, study design, location and period, sampling technique, sample size, number of cases, diagnostic criteria, type of examiners, number decayed, missing, and filled teeth (dmft). Where possible, each category was sub-grouped according to the year of publication, age, urban/rural area, and Province. If it was unclear whether the area was urban or rural, the information was designated to an “urban/rural” category.

### Critical appraisal

The studies’ quality was assessed by two independent authors (FK and TR) using the Joanna Briggs Institute (JBI) Critical Appraisal Checklist for Studies Reporting Prevalence Data [22, 23]. The specific JBI Critical Appraisal tool contained nine explicit criteria, and a maximum score of nine indicated the lowest risk of bias, Table 1. The process was repeated twice by the same authors. Any inconsistencies which arose between the two authors were resolved by consulting with the remaining authors. Two independent authors (FK and TR) judged the scoring, and a final decision was reached by consensus with all the authors.

**Table 1:** Critical Appraisal of included studies

First author, year	Age	Was the sample frame appropriate to address the target population?	Were study participants recruited in an appropriate way?	Was the sample size adequate?	Were the study subjects and settings described in detail?	Was data analysis conducted with sufficient coverage of the identified sample?	Were valid methods used for the identification of the condition?	Was the condition measure din a standard, reliable way for all the participants?	Was there appropriate statistical analysis?	Was the response rate adequate, and if not, was the low response rate managed appropriately?	Total
Booyens (1991) [21]	5	Yes	Yes	Yes	No	Yes	Yes	Yes	No	Yes	7
Brindle (2000) [22]	5–6	Yes	Yes	Yes	Yes	Yes	Yes	Yes	No	Yes	8
Chosack (1988) [24]	3–5	No	No	No	Yes	Yes	Yes	Yes	Yes	Yes	6
Chosack (1990) [25]	3–5	Yes	Yes	No	No	Yes	Yes	Yes	No	Yes	6
Cleaton-Jones (1984) [26]	1–5	No	No	Yes	No	No	Yes	Yes	No	Yes	4
Cleaton-Jones (1978) [27]	1–5	No	No	Yes	No	No	Yes	No	No	Yes	3
Cleaton-Jones (1978) [28]	1–5	Yes	No	Yes	Yes	No	Yes	No	No	Yes	5
Cleaton-Jones (1981) [29]	1–5	No	Yes	Yes	Yes	No	Yes	Yes	No	Yes	6
Cleaton-Jones (1989) [30]	1–4	No	Yes	Yes	Yes	Yes	Yes	Yes	No	Yes	7
Cleaton-Jones (2000) [31]	2–5	Yes	Yes	Yes	Yes	No	Yes	Yes	Yes	Yes	8
Cleaton-Jones(2008) [32]	2–5	Yes	Yes	Yes	Yes	No	Yes	Yes	Yes	No	7
Du Plessis (2000) [33]	4–5	Yes	No	No	Yes	Yes	Yes	Yes	No	Yes	6
Gordon 1985) [34]	1–2	No	No	No	No	No	Yes	Yes	No	Yes	3
Gordon (2007) [35]	< 6	Yes	No	No	No	No	No	No	No	No	1
Granath 1991) [36]	4–5	Yes	Yes	Yes	Yes	Yes	Yes	Yes	Yes	Yes	9
Granath (1993) [37]	4	Yes	Yes	Yes	Yes	Yes	Yes	Yes	Yes	No	8
Khan (1998) [38]	3–5	No	Yes	Yes	Yes	Yes	Yes	Yes	Yes	Yes	8
McInnes (1979) [39]	3–5	Yes	No	No	No	Yes	Yes	No	No	Yes	4
Mndzebele (2014) [40]	2–6	Yes	Yes	No	Yes	No	Yes	Yes	Yes	Yes	7
Mohamed (2018) [41]	1–5	Yes	No	Yes	Yes	Yes	Yes	No	No	Yes	6
Mothupi (2016) [42]	4–6	Yes	Yes	Yes	Yes	No	Yes	Yes	Yes	Yes	8
Ntombela (2015) [43]	1–5	Yes	Yes	Yes	Yes	Yes	Yes	No	Yes	Yes	8
Richardson (1978) [44]	1–6	No	Yes	Yes	No	No	No	No	No	Yes	3
Roberts (1993) [45]	1–4	No	Yes	Yes	No	Yes	Yes	Yes	Yes	Yes	7
Thekiso (2012) [46]	4–5	Yes	Yes	Yes	Yes	Yes	Yes	Yes	No	Yes	8
Toi (1999) [47]	5	No	No	No	Yes	Yes	Yes	Yes	Yes	Yes	6
Van Wyk (2004a) [9]	4–6	Yes	Yes	Yes	Yes	Yes	Yes	Yes	No	Yes	8
Wanjau (2006) [48]	3–5	Yes	Yes	Yes	Yes	Yes	Yes	Yes	No	Yes	8
Williams (1985) [49]	2–5	No	No	Yes	Yes	No	Yes	Yes	No	Yes	5

### Data synthesis

Meta-analyses were conducted using StataCorp. 2019. STATA Statistical Software: Release 17, College Station, TX: StataCorp LLC. The pooled estimates and 95% confidence intervals for each indicator were calculated by combining each study’s data. Q-test and I^2^-statistical analysis were used to determine statistical heterogeneity. A random-effects model was adopted because of significant heterogeneity (I^2^ > 50%); Subgroup analysis was conducted to explore possible factors, including urban and rural status, age distribution and Province.

To reflect ECC’s spatial distribution, pooled prevalence estimates for ECC in all children under six years in each Province during 1975–2014 were entered into the QGIS software 3.8.3 (2019) to form a prevalence map.

### Patient and public involvement statement

Neither patients nor the public was involved in the design, conduct, reporting, or dissemination of research plans.

## Results

### Search and selection results

A total of 2441 publications were identified in the search strategy, and a further seven were identified through other sources. After 194 articles were removed due to duplication, 2254 articles were analysed.

**Fig. 1:**
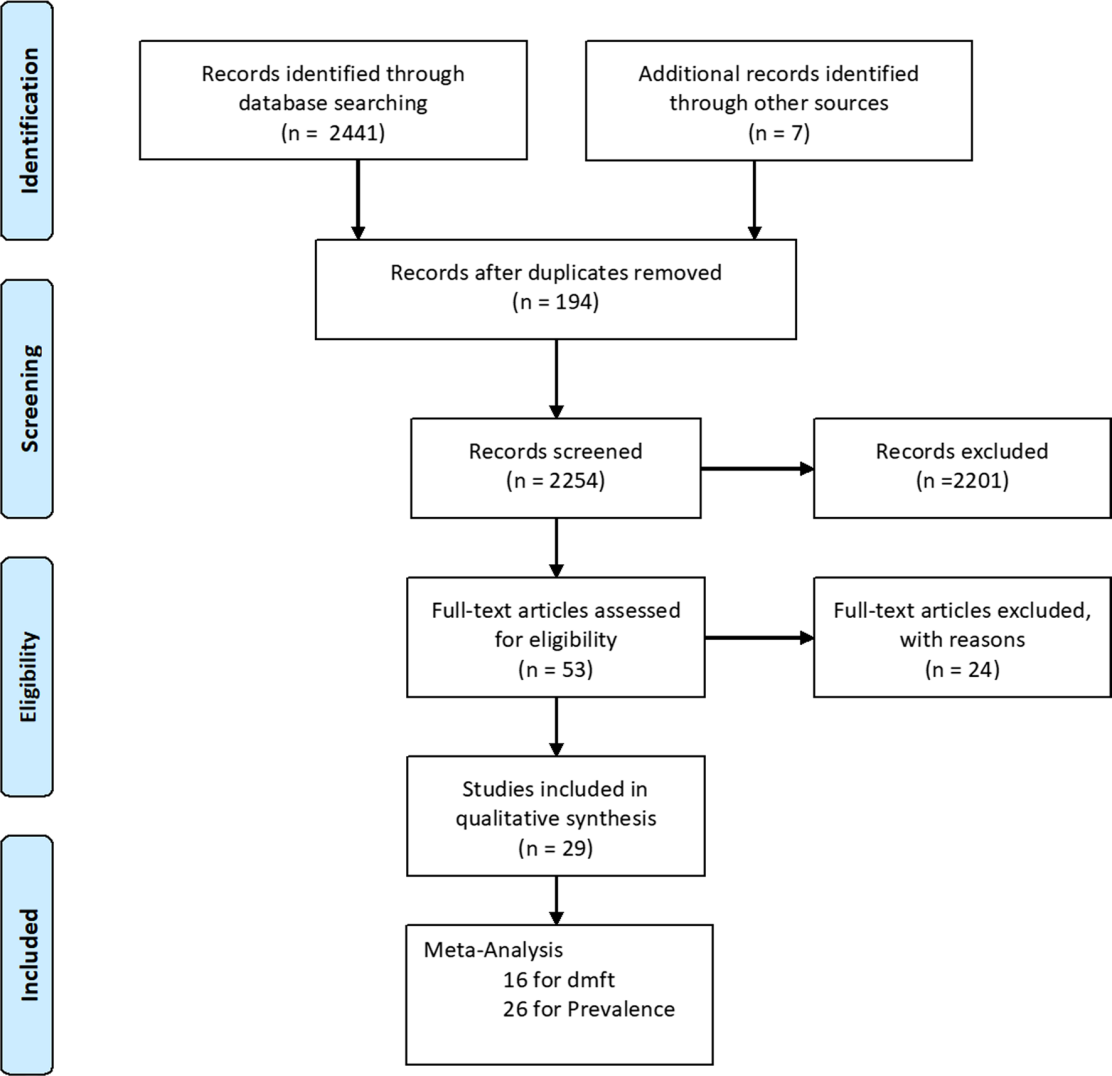
Flow chart of literature search and selection

After reading the titles and abstracts of the remaining articles, 2201 were excluded, and two authors independently evaluated the remaining 53 full-text articles for eligibility. After the full-text analysis, 24 were excluded because they did not meet the inclusion criteria, and 29 were included in the meta-analysis (Fig. 1, Table 2). Thus, the total sample size was 29,477 individuals. The characteristics of the 29 studies are summarised in Table 1. Sixteen articles had information on the dmft score, and 26 had information on prevalence. The overall prevalence was 44.94% (Table 3), and the overall dmft score was 2.422 (Table 4). Of these, 28 studies used diagnostic criteria established by the World Health Organization (WHO) Oral Basic Surveys Methods 2,3 and 4. In 12 studies, dentists examined the participants (Table 5). Characteristics of included studies can be found in Table 5.

**Table 2:** Table of excluded studies

Title	Reason for exclusion
Caries and dental hygiene [50]	Review
Caries and micronutrient intake among urban South African children: a cohort study [51]	No dmft/prevalence stated
Dental caries at five and twelve years in a South African Indian community: a longitudinal study [52]	No dmft/prevalence stated
Dental caries in Black preschool children in Cape Town [53]	No sd for dmft and prevalence data was pooled for 4–7 year olds
Dietary intakes and caries experience in children in Limpopo Province, South Africa. [54]	Wrong population
Epidemiological profile of patients utilising public oral health services in Limpopo province, South Africa [55]	Data was pooled for age 6–10
Occlusal and oral health status of a group of 3–8-year-old South African black children. [56]	Combined data
Oral health status, knowledge, attitude and behaviour of Riverlea Primary School children [57]	No dmft data and prevalences were pooled
Oral health in South Africa [58]	Review
Patterns of breast and bottle feeding and their association with dental caries in 1- to 4-year-old South African children 2. A case control study of children with nursing caries [59]	Not primary data
Prevalence of dental caries in !Kung Bushmen of Bushmanland [60]	Pooled prevalence data
Prevalence of dental caries in preschool and primary school children in Mamre [61]	Wrong outcome
Priority health problems of children in an urban community [62]	No dental data
Socio-demographic correlates of early childhood caries prevalence and severity in a developing country—South Africa [63]	Not primary data
The impact or urbanisation on the health of black pre-school children in the Umtata district, Transkei, 1990 [64]	No dental data
The prevalence of oral pathoses in a private dental practice: a 30 month survey. (Bernitz et al*.* 1998) [65]	Wrong outcome
The relationship between the intake frequency and the total consumption of sucrose among four South African ethnic groups [66]	No dental data
Trends in dental caries prevalence, severity and unmet treatment need levels in South Africa between 1983 and 2002 [67]	Not a primary study
Trends in sugar intake: do these parallel changes in caries prevalence among S. African preschoolchildren? [68]	Review
Urbanisation and cariogenic food habits among 4–24-month-old black South African children in rural and urban areas [69]	No dental data
Dental caries in six, 12 and 15 year old Venda children in South Africa [70]	Wrong population
Dental status of rural school children in a sub-optimal fluoride area [71]	Wrong population
The burden of dental caries in the Western Cape and a recommended turn-around strategy [9]	Wrong population
The effect of socio-economic status on dental caries experience in 6, 12 and 15 year-old school children in Port Elizabeth and Despatch [72]	Wrong population

**Table 3:** Pooled prevalence per age group, year period and urban or rural status in South Africa from 1978–2019

	Number of studies	Cases	Sample size	Random pooled prevalance	95% CI (%)
Overall		13,904	29,477	44.94	39.73–50.15
Age					
1	5	105	731	21.48	0.090–33.95
2	9	670	2130	31.71	24.49–38.92
3	12	1360	3575	43.37	31.89–54.84
4	14	3592	7067	49.58	38.49–60.67
5	14	2205	5159	51.72	36.67–66.78
< 6	9	5972	11,143	52.60	46.58–58.61
Year period					
1978–1979	6	2115	3622	57.37	46.48–68.25
1980–1984	3	3234	6398	39.31	28.39–50.24
1985–1989	4	1941	4967	36.38	21.70–45.65
1990–1994	2	743	3230	23.08	14.59–1.57
1995–1999	5	4423	9029	40.80	32.14–49.47
2000–2004	2	223	442	48.38	34.63–60.33
2005–2009	1	129	269	45.04	26.89–63.19
2010–2014	5	1096	1848	61.75	52.93–70.57
Urban or rural					
Rural	8	1344	2780	47.40	41.13–53.67
Urban	16	7274	16,543	43.72	36.56–50.89
Urban/rural	3	5286	10,482	48.22	37.80–58.63
Province					
Limpopo	2	282	733	37.36	33.90–40.82
Gauteng	21	9901	22,297	43.10	37.01–49.20
Kwazulu Natal	2	1069	2017	53.04	50.87–55.22
Western Cape	2	1166	1649	73.39	66.59–80.19
Eastern Cape	1	122	228	53.51	47.03–59.87
Free State	1	532	885	60.11	56.85–63.29
North West	1	418	1019	41.02	38.04–44.07
Mpumalanga	2	414	977	43.76	32.85–54.66

**Table 4:** Pooled dmft score by age, year period and urban and rural status in South Africa from 1978—2019

	Number of studies	Effect size (mean)	95% CI (%)
Overall dmft		2.422	2.148–2.696
Age			
1	4	1.027	0.000–2.122
2	7	1.048	0.820–1.277
3	9	1.963	1.695–2.322
4	10	2.743	2.422–3.064
5	11	3.485	3.055–3.915
< 6	4	3.404	1.476–5.332
Year period			
1978–1979	4	2.957	2.267–3.648
1980–1984	2	2.012	1.538–2.486
1985–1989	5	2.448	2.121–2.855
1990–1994	1	1.849	1.264–2.434
1995–1999	2	1.042	0.447–1.638
2000–2004	2	2.315	1.564–3.067
2005–2009	–	–	–
2010–2014	2	4.068	0.682–7.455
Urban or rural			
Rural	4	2.185	1.272–3.098
Urban	13	2.390	2.094–2.685
Urban/rural	1	4.260	2.753–5.767
Province			
Limpopo	1	0.330	0.066–0.594
Gauteng	13	2.442	2.163–2.722
Kwazulu Natal	1	3.000	2.863–3.317
Western Cape	1	2.370	1.996–2.744
Eastern Cape	1	3.850	3.454–4.246
Northern Cape	–	–	–
Free State	–	–	–
North West	–	–	–
Mpumalanga	–	–	–

**Table 5:** Table of included studies

First author, year	Year survey (if different from publication date)	Age	OH/dentist	Sampling technique	WHO/ICDAS	Area	Urban/rural
Booyens (1991) [21]	1987	5	Unclear	Random Cluster Sampling	WHO 1977	Pretoria	Urban
Brindle (2000) [22]	2000	5–6	Unclear	Random Cluster Sampling	WHO, III, 1997	Kwazulu Natal	Rural
Chosack (1988) [24]	1988	3–5	Unclear	Convenience Sampling Technique	WHO 1977	Unclear	Unclear
Chosack (1990) [25]	1990	3–5	Dental Epidemiologist	Convenience Sampling Technique	WHO 1979	Gauteng	Urban
Cleaton-Jones (1984) [26]	1981	1–5	Unclear	Convenience Sampling Technique	?WHO,II,1977	Gauteng	Rural
Cleaton-Jones (1978) [27]	1978	1–5	Dentist	?Cluster random Sampling Technique	?WHO,II,1977	Gauteng	Urban
Cleaton-Jones (1978) [28]	1978	1–5	Dentist	Convenience Sampling Technique	?WHO,II,1977	Gauteng	Urban/Rural
Cleaton-Jones (1981) [29]	1981	1–5	Unclear	Convenience Sampling Technique	?WHO,II,1977	Gauteng	Urban
Cleaton-Jones (1989) [30]	1985	1–4	Unclear	Simple random sampling technique	WHO,II, 1977	Limpopo	Rural
Cleaton-Jones (2000) [31]	1981–1997	2–5	Dentist	Convenience Sampling Technique	WHO, IV, 1997	Gauteng	Urban
Cleaton-Jones (2008) [32]	1981–2002	2–5	Dentist	Convenience Sampling Technique	Unclear	Gauteng	Urban
Du Plessis (2000) [33]	2000	4–5	Unclear	Convenience Sampling Technique	WHO, 1987	Rural	Northern Province
Gordon (1985) [34]	1985	1–2	Unclear	Convenience Sampling Technique	?WHO, 1977	Western Cape	Urban
Gordon (2007) [35]	2007	1–2	Oral Hygienist	Convenience Sampling Technique	WHO, 1997	Western Cape	Urban
Granath (1991) [36]	1984	4–5	Unclear	Convenience sampling	WHO II, 1977	Gauteng	Urban/Rural
Granath (1993) [37]	1984	4–5	Unclear	Simple random sampling technique	WHO II, 1997	Gauteng	Urban/Rural
Khan (1998) [38]	1998	3–5	Dentist	Convenience sampling	WHO, III	Gauteng	Rural
McInnes (1979) [39]	1978	3–5	Unclear	Convenience Sampling Technique	?WHO, 1977	Gauteng	Urban
Mndzebele (2014) [40]	2010	2–6	Unclear	Convenience sampling	WHO, III	Gauteng	Urban
Mohamed (2018) [41]	2016	1–5	Dentist	Convenience sampling	ICDAS	Western Cape Province	Urban
Mothupi (2016) [42]	2013	4–6	Unclear	Stratified Random Sampling	WHO, 1997	Gauteng	Urban
Ntombela (2015) [43]	2014	1–5	Unclear	simple random stratification	?WHO, 1997	Gauteng	Urban
Richardson (1978) [44]	1978	1–6	Dentist	Convenience sampling	WHO, 1971	Gauteng	Urban/Rural
Roberts (1993) [45]	1993	1–4	Unclear	Convenience sampling	WHO, 1979	Unclear? Gauteng	?Urban
Thekiso (2012) [46]	2010	4–5	Oral Health Professional	Cluster randomised sampling	WHO, 1979	Gauteng	Urban
Toi (1999) [47]		5	Dentist	Convenience sampling	WHO, 1987	Gauteng	Urban
Van Wyk (2004) [9]	1999	4–6	Dentist	Cluster randomised sampling	WHO, IV	National Survey	Urban/Rural
Wanjau (2006) [48]	2006	3–5	Unclear	Cluster randomised sampling	WHO 1997	Mpumalanga	Rural
Williams (1985) [49]	1981–1983	2–5	Dentist	Convenience Sampling Technique	WHO, 1977	Gauteng	Urban

### Prevalence of ECC in South Africa

The pooled overall prevalence of ECC was 44.94% (95% C.I. 39.73–50.15%). The prevalence for 4- and 5- year-olds were reported in 14 studies. ECC’s overall prevalence ranged from 57.37% between 1975 and 1979 to 61.75% between 2010 and 2014, indicating a U-shaped trend over time. The rural prevalence was higher than the urban prevalence of ECC.

**Fig. 2:**
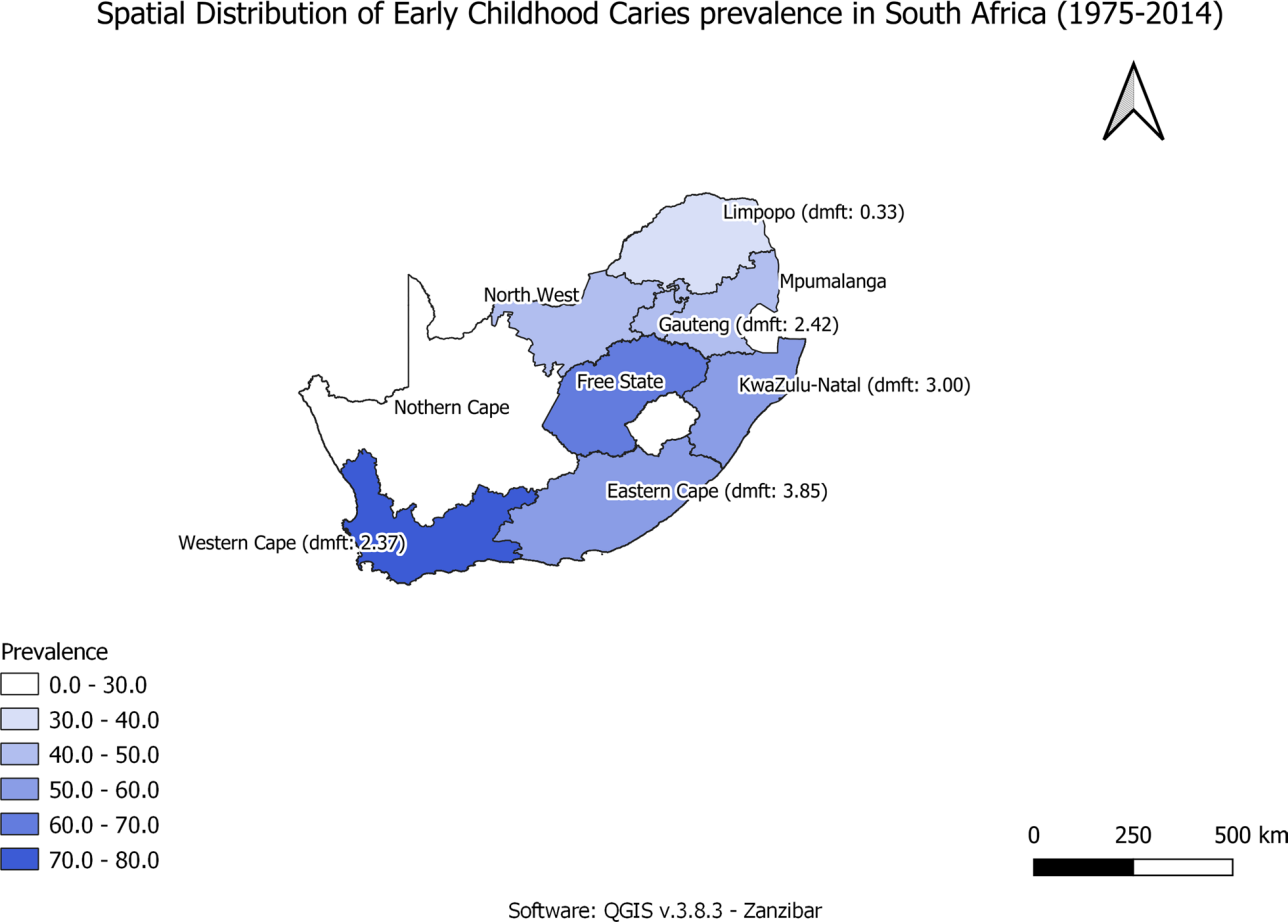
Spatial Distribution of ECC prevalence and dmft scores in South Africa

The overall prevalence of dental caries by Province is illustrated in Fig. 2. The prevalence of dental caries decreased from 1975 to 1994 but showed an increase from 1995 to the present, Table 1.

**Fig. 3:**
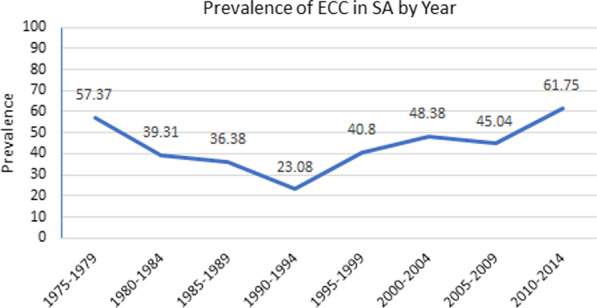
Prevalence of ECC in South Africa by Year

The dmft per Province was 3.850, 3.000, 2.370, 2.442 and 0.330 in Eastern Cape, Kwazulu Natal, Western Cape, Gauteng, and Limpopo Provinces, respectively (Fig. 3).

#### Publication bias

Funnel plots and Begg’s test assessed potential publication bias; the result was significant if *p* ≤ 0.05.

Duval and Tweedie’s "Trim and Fill" method was used to assess publication bias for the prevalence and dmft scores. Under the random-effects model, the point estimate for prevalence and 95% confidence interval for the pooled was 45.7% (44.8–46.7%). Using Trim and Fill, the imputed point estimate was the same. The method suggests a total of 0 studies missing from this review for the prevalence estimate.

**Fig. 4:**
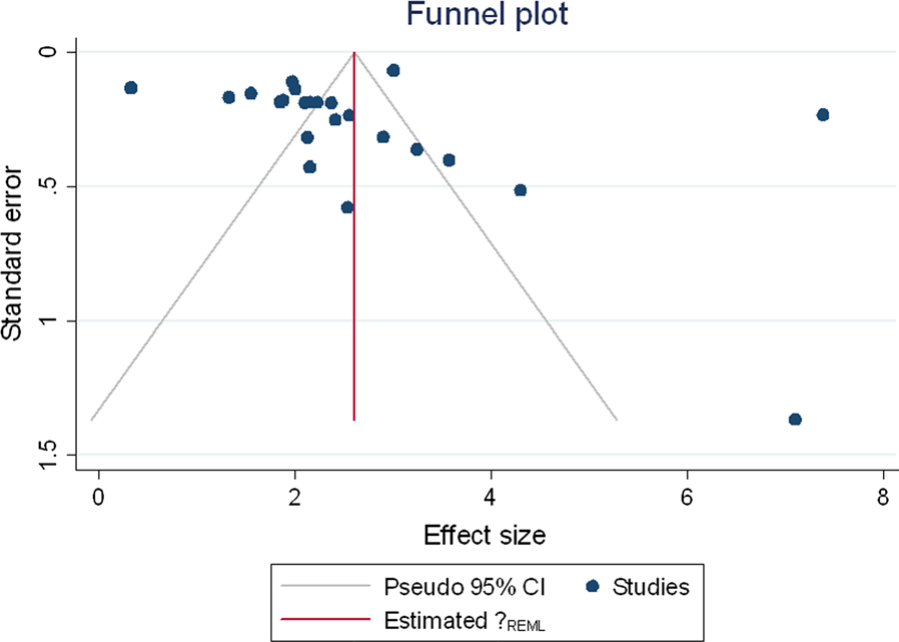
Publication bias dmft score

**Fig. 5:**
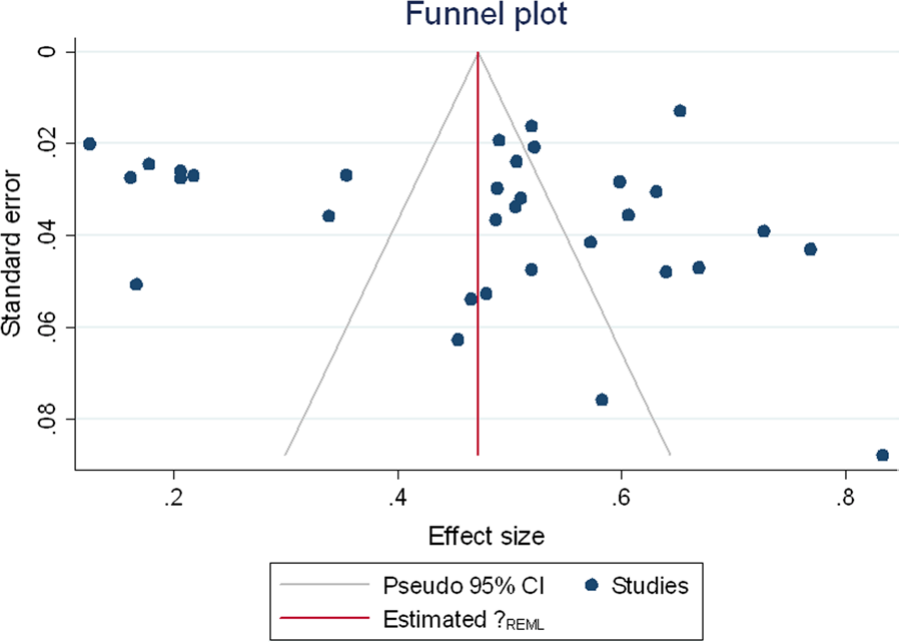
Publication bias prevalence estimate

In addition, there were no missing studies for the dmft score as the pooled point estimate was 2.307 (2.236–2.378) and using Trim and Fill, these values remain unchanged. Egger's test results were significant for both dmft, *p* < 0.001 (Fig. 4) and prevalence, *p* = 0.0031 (Fig. 5). These results suggest that there was publication bias.

## Discussion

### Summary of main findings

The oral and dental health of individuals are essential to general health, and even more so in children. The current investigation is the first systematic review on the prevalence and severity of ECC in South Africa. The results summarise the last 30 years of prevalence studies among children under 71 months in South Africa.

### Agreements and disagreements with previous studies

The meta-analyses of the observational data collected from the eligible studies in the current study have provided a summary estimate of ECC’s prevalence in South Africa. The overall pooled prevalence of ECC was 44.94% and 51.72% for 5-year-old children. The figures are much lower than other middle to upper-income countries, including Albania, 84.0% [73], American Samoa 87.0% [74], Argentina, 80.4% [75], and Turkey, 70.5% [76]. However, they are much higher than that of Namibia [77], which has a prevalence of 31.69%.

The prevalence of dental caries increased as age increased. This corroboratesthe findings from a systematic review of the prevalence of early childhood caries in China [78]. Caries prevalence seems to have decreased from the 1970’s till the early 1990’s, Table 1. Thereafter, the prevalence of caries in children under 6 appears to increase over time. After 1994, the South African government aimed to improve the living standards by providing the poor with housing rather than providing the poor with higher incomes [79]. There was thus an increase in housing and infrastructure but, no change in employment [79]. Furthermore, there has also been an increase in urbanisation. The majority of the population in South Africa is Black, and under the apartheid regime, they were restricted in their mobility [ability to move to urban areas for work]. After the apartheid laws were revoked in June 1991 there was an increase in mobility towards the cities. Post-Apartheid urbanisation has resulted in greater access to sugar and junk food compared to rural areas [80]. This may have resulted in the higher caries prevalence noted post-apartheid.

Of interest was the declining trend in the prevalence of caries prevalence as one moved to the north of the country. The National Children's Oral Health Survey, 2001–2002, indicated that the Limpopo Province had the lowest rate of dental caries in children: 31.30% of 4–5-year-olds and 30.80% in 6-year-old children [9]. The Limpopo Province is one of the poorest regions of South Africa, with a large disparity between poor and affluent residents, especially in the rural areas [81]. Limpopo Province is an arid land, and 75.00% of the population is dependent on groundwater. The Limpopo and North-West provinces have been identified as having high fluoride levels, up to 30 mg/l [82] and they also present with the lowest prevalence of dental caries at 37.36% and 41.02%, respectively.

A challenge or limitation of the current review is that most of the studies were conducted in the Western Cape and Gauteng Provinces. The authors strongly suggest that examiners are thoroughly trained and that test–retest validity is conducted in all future prevalence studies in South Africa. It would also be favourable that a single tool (standardised) be used to examine dental prevalence. The choice of dental disease tool should be based on the exact outcome of the study performed. While the dmft score is sufficient for a dental prevalence and severity study, pulpal involvement, ulceration, fistula and abscess (pufa) score is better suited to determine the severity of clinical outcomes related to the dental treatment needs of the study under investigation.

Caution should be exercised when evaluating the current study results as there is a high heterogeneity among the included studies; this is not uncommon when evaluating systematic reviews of this nature.

## Supplementary Information

**Additional file 1:** This is the completed MOOSE Checklist for Meta-analyses of Observational Studies completed for this paper.

**Additional file 2:** Characteristics of included studies consisting of the raw data such author, sample size and mean and SD of dmft.

## Data Availability

The datasets generated and/or analysed during the current study are available from the kikapu repository, https://doi.org/10.25379/uwc.14873340.v1.

## Abbreviations

ECC: Early childhood caries
NCDs: Non communicable diseases
dmft: decayed, missing, and filled teeth
JBI: Joanna Briggs Institute
MOOSE: Meta-analysis of Observational Studies in Epidemiology
