# Supplementary material for: Prevalence of early childhood caries in South Africa: a systematic review
Source: BMC Oral Health. 2022 Feb 8;22:32. doi: 10.1186/s12903-021-01982-6 (PMC10074718; doi:10.1186/s12903-021-01982-6)
Supplement: Supplementary file 2 — Additional file 2: Characteristics of included studies consisting of the raw data such author, sample size and mean and SD of dmft. [file 12903_2021_1982_MOESM2_ESM.pdf]

Supplementary Table S2: Characteristics of included studies

Author (year)	Year, if different from publication date	Collection period	AGE	n	N	dmft (mean)	dmft (SD)	Urban/Rural	Province
Bajomo (2004) [23]	1997	1995-1999	6	63	94	3.41	3.8	Urban/Rural	Limpopo
Bajomo (2004) [23]	1997	1995-1999	6	52	92	1.93	2.4	Rural	Limpopo
Booyens (1991) [24]	1987	1985-1989	5	329	670	1.97	2.87	Urban	Gauteng
Brindle (2000) [25]	2000	2000-2004	5-6	64	100	3	0.7	Rural	Kwazulu Natal
Carstens (1995) [26]	1993	1990-1994	6	24	39	3.31	0.08	Rural	Western Cape
Chosack (1988) [27]	1988	1985-1989	3		69	2.93	3.63	Urban/Rural	Gauteng
Chosack (1988) [27]	1988	1985-1989	4		82	4.37	4.54	Urban/Rural	Gauteng
Chosack (1988) [27]	1988	1985-1989	5		77	5.61	5.32	Urban/Rural	Gauteng
Chosack (1990) [28]	1986	1985-1989	3	29	51	3	4.3	Urban	Gauteng
Chosack (1990) [28]	1986	1985-1989	4	20	41	2.3	3.6	Urban	Gauteng
Chosack (1990) [28]	1986	1985-1989	5	25	35	2.3	3.4	Urban	Gauteng
Cleaton Jones (1984) [29]	1981	1975-1979	2	21	41	2.1	3.1	Rural	Gauteng
Cleaton Jones (1984) [29]	1981	1975-1979	3	20	37	3.6	4.6	Rural	Gauteng
Cleaton Jones (1984) [29]	1981	1975-1979	4	29	37	3.7	3.6	Rural	Gauteng
Cleaton Jones (1984) [29]	1981	1975-1979	5	17	24	4.3	4.6	Rural	Gauteng
Cleaton Jones (1984) [29]	1981	1975-1979	2	75	159	1.19	2.25	Urban	Gauteng

Cleaton Jones (1984) [29]	1981	1975-1979	3	149	182	2.17	3.72	Urban	Gauteng
Cleaton Jones (1984) [29]	1981	1975-1979	4	153	160	4.73	4.53	Urban	Gauteng
Cleaton Jones (1984) [29]	1981	1975-1979	5	125	126	6.75	5.17	Urban	Gauteng
Cleaton-Jones (1978) [31]	1978	1975-1979	1	6	50	0.6	2	Rural	Gauteng
Cleaton-Jones (1978) [31]	1978	1975-1979	2	21	70	1.5	2.9	Rural	Gauteng
Cleaton-Jones (1978) [31]	1978	1975-1979	3	39	92	1	3.2	Rural	Gauteng
Cleaton-Jones (1978) [31]	1978	1975-1979	4	53	126	1.9	3.2	Rural	Gauteng
Cleaton-Jones (1978) [31]	1978	1975-1979	5	51	101	3	4.2	Rural	Gauteng
Cleaton-Jones (1978) [31]	1978	1975-1979	1	1	6	1	2.4	Urban	Gauteng
Cleaton-Jones (1978) [31]	1978	1975-1979	2	13	62	1.3	3.3	Urban	Gauteng
Cleaton-Jones (1978) [31]	1978	1975-1979	3	54	57	2.7	4	Urban	Gauteng
Cleaton-Jones (1978) [31]	1978	1975-1979	4	27	38	4.1	4.1	Urban	Gauteng
Cleaton-Jones (1978) [31]	1978	1975-1979	5	21	29	4.4	4.8	Urban	Gauteng
Cleaton-Jones (1981) [32]	1981	1975-1979	1	8	102	0.16	0.79	Urban	Gauteng
Cleaton-Jones (1981) [32]	1981	1975-1979	2	30	96	1.3	2.82	Urban	Gauteng
Cleaton-Jones (1981) [32]	1981	1975-1979	3	52	102	2.57	3.77	Urban	Gauteng
Cleaton-Jones (1981) [32]	1981	1975-1979	4	93	126	4.41	4.5	Urban	Gauteng
Cleaton-Jones (1981) [32]	1981	1975-1979	5	105	128	6.04	4.83	Urban	Gauteng
Cleaton-Jones (1978) [30]	1978	1975-1979	1	12	32	1	2	Urban	Gauteng
Cleaton-Jones (1978) [30]	1978	1975-1979	2	52	98	2.5	3.6	Urban	Gauteng
Cleaton-Jones (1978) [30]	1978	1975-1979	3	90	114	3.9	3.9	Urban	Gauteng

Cleaton-Jones (1978) [30]	1978	1975-1979	4	92	136	3.7	4.2	Urban	Gauteng
Cleaton-Jones (1978) [30]	1978	1975-1979	5	88	119	5.1	4.5	Urban	Gauteng
Cleaton-Jones (1989)	1985	1985-1989	1,2,3,4	911	1436			Urban/Rural	Gauteng
Cleaton-Jones (2000) [34]	2000	1980-1984	2	28	100	0.9	1.9	Urban	Gauteng
Cleaton-Jones (2000) [34]	1983	1980-1984	2	26	100	0.7	1.4	Urban	Gauteng
Cleaton-Jones (2000) [34]	1985	1985-1989	2	24	93	0.8	1.9	Urban	Gauteng
Cleaton-Jones (2000) [34]	1987	1985-1989	2	32	104	1.1	2.3	Urban	Gauteng
Cleaton-Jones (2000) [34]	1989	1985-1989	2	39	90	1.3	2.3	Urban	Gauteng
Cleaton-Jones (2000) [34]	1991	1990-1994	2	19	198	0.7	2	Urban	Gauteng
Cleaton-Jones (2000) [34]	1994	1995-1999	2	12	87	0.7	1.4	Urban	Gauteng
Cleaton-Jones (2000) [34]	1997	1995-1999	2	8	79	0.2	0.9	Urban	Gauteng
Cleaton-Jones (2000) [34]	2000	1980-1984	3	50	230	1.7	2.6	Urban	Gauteng
Cleaton-Jones (2000) [34]	1983	1980-1984	3	40	205	1.5	2.7	Urban	Gauteng
Cleaton-Jones (2000) [34]	1985	1985-1989	3	44	184	1.5	2.6	Urban	Gauteng
Cleaton-Jones (2000) [34]	1987	1985-1989	3	47	221	2	3	Urban	Gauteng
Cleaton-Jones (2000) [34]	1989	1985-1989	3	60	148	2.6	3.2	Urban	Gauteng
Cleaton-Jones (2000) [34]	1991	1990-1994	3	39	254	1.5	2.7	Urban	Gauteng
Cleaton-Jones (2000) [34]	1994	1990-1994	3	29	272	1.2	2.4	Urban	Gauteng
Cleaton-Jones (2000) [34]	1997	1995-1999	3	27	192	1.3	2.5	Urban	Gauteng
Cleaton-Jones (2000) [34]	2000	1980-1984	4	53	291	2.2	3.1	Urban	Gauteng
Cleaton-Jones (2000) [34]	1983	1980-1984	4	53	274	2.4	3.3	Urban	Gauteng

Cleaton-Jones (2000) [34]	1985	1985-1989	4	58	228	2.7	3.6	Urban	Gauteng
Cleaton-Jones (2000) [34]	1987	1985-1989	4	57	300	2.3	3	Urban	Gauteng
Cleaton-Jones (2000) [34]	1989	1985-1989	4	66	235	3.2	3.7	Urban	Gauteng
Cleaton-Jones (2000) [34]	1991	1990-1994	4	55	244	2.2	3	Urban	Gauteng
Cleaton-Jones (2000) [34]	1994	1990-1994	4	43	331	1.9	3.1	Urban	Gauteng
Cleaton-Jones (2000) [34]	1997	1995-1999	4	35	210	1.6	2.6	Urban	Gauteng
Cleaton-Jones (2000) [34]	2000	1980-1984	5	72	312	3.8	3.9	Urban	Gauteng
Cleaton-Jones (2000) [34]	1983	1980-1984	5	59	284	2.8	3.6	Urban	Gauteng
Cleaton-Jones (2000) [34]	1985	1985-1989	5	66	237	3	3.5	Urban	Gauteng
Cleaton-Jones (2000) [34]	1987	1985-1989	5	63	341	3	3.5	Urban	Gauteng
Cleaton-Jones (2000) [34]	1989	1985-1989	5	71	225	3.1	3.3	Urban	Gauteng
Cleaton-Jones (2000) [34]	1991	1990-1994	5	60	277	3.1	3.5	Urban	Gauteng
Cleaton-Jones (2000) [34]	1994	1990-1994	5	51	391	2.4	3.3	Urban	Gauteng
Cleaton-Jones (2000) [34]	1997	1995-1999	5	46	238	2.2	3.1	Urban	Gauteng
Cleaton-Jones (2008) [35]	2002	2000-2004	2	23	64	1.5	2.3	Urban	Gauteng
Cleaton-Jones (2008) [35]	2002	2000-2004	3	27	78	1.6	2.9	Urban	Gauteng
Cleaton-Jones (2008) [35]	2002	2000-2004	4	35	76	2	2.9	Urban	Gauteng
Cleaton-Jones (2008) [35]	2002	2000-2004	5	74	124	3.4	3.7	Urban	Gauteng
Du Plessis (1997) [36]	1997	1995-1999	6		299	3.85	3.49	Urban/Rural	Eastern Cape
Du Plessis (2000) [37]	1997	1995-1999	4-5	9	54	0.33	0.99	Rural	Limpopo
Du Plessis (2000) [37]	1997	1995-1999	6	13	52	0.67	1.39	Rural	Limpopo

Gordon (1985) [38]	1985	1985-1989	1		100	2.37	1.91	Urban	Western Cape
Gordon (2007) [39]	1985	1985-1989	1	12	49			Urban	Western Cape
Granath (1991) [40]	1984	1980-1984	4	345	671			Rural	Gauteng
Granath (1991) [40]	1984	1980-1984	4	1435	2057			Urban	Gauteng
Granath (1993) [41]	1984	1980-1984	4-5	1689	2576			Urban/Rural	Gauteng
Khan (1998) [42]	1998	1995-1999	3	43	91			Rural	Gauteng
Khan (1998) [42]	1998	1995-1999	4	109	189			Rural	Gauteng
Khan (1998) [42]	1998	1995-1999	5	92	146			Rural	Gauteng
McInnes (1979) [43]	1979	1975-1979	3-5	15	18	7.1	5.8	Urban	Gauteng
Mndzebele (2014) [44]	2010	2010-2014	<6	125	245			Rural	Gauteng
Mohamed (2018) [45]	2014	2010-2014	1	52	103			Urban	Western Cape
Mohamed (2018) [45]	2014	2010-2014	2	87	129			Urban	Western Cape
Mohamed (2018) [45]	2014	2010-2014	3	134	172			Urban	Western Cape
Mohamed (2018) [45]	2014	2010-2014	4	113	142			Urban	Western Cape
Mohamed (2018) [45]	2014	2010-2014	5	86	103			Urban	Western Cape
Mothupi (2016) [46]	2013	2010-2014	4	68	135	2.49	3.51	Urban	Gauteng
Mothupi (2016) [46]	2013	2010-2014	5	114	238	2.33	3.41	Urban	Gauteng
Mothupi (2016) [46]	2013	2010-2014	6	44	86	2.02	3.05	Urban	Gauteng
Ntombela (2015) [47]	2014	2010-2014	<6	179	299	7.38	4.06	Urban	Gauteng
Richardson (1978) [48]	1978	1975-1979	<6	171	437			Rural	Gauteng
Richardson (1978) [48]	1978	1975-1979	<6	432	717			Urban	Gauteng

Roberts (1993) [49]	1993	1990-1994	1	26	338			Urban/Rural	Gauteng
Roberts (1993) [49]	1993	1990-1994	2	106	360			Urban/Rural	Gauteng
Roberts (1993) [49]	1993	1990-1994	3	164	317			Urban/Rural	Gauteng
Roberts (1993) [49]	1993	1990-1994	4	151	248			Urban/Rural	Gauteng
Smit (2017) [12]	2011-2015	2015-2019	6	1473	1754			Urban/Rural	Western Cape
Thekiso (2012) [50]	2010	2010-2014	4-5	138	282			Urban	Gauteng
Toi (1999) [51]	1999	1995-1999	5	114	188			Urban	Gauteng
Van Wyk (2004) [11]	1999	1995-1999	4-5	694	900			Urban/Rural	Western Cape
Van Wyk (2004) [11]	1999	1995-1999	6	647	899			Urban/Rural	Western Cape
Van Wyk (2004) [11]	1999	1995-1999	4-5	122	228			Urban/Rural	Eastern Cape
Van Wyk (2004) [11]	1999	1995-1999	6	135	199			Urban/Rural	Eastern Cape
Van Wyk (2004) [11]	1999	1995-1999	6	264	366			Urban/Rural	Northern Cape
Van Wyk (2004) [11]	1999	1995-1999	4-5	532	885			Urban/Rural	Free State
Van Wyk (2004) [11]	1999	1995-1999	6	540	913			Urban/Rural	Free State
Van Wyk (2004) [11]	1999	1995-1999	4-5	1005	1917			Urban/Rural	Kwazulu Natal
Van Wyk (2004) [11]	1999	1995-1999	6	1235	1906			Urban/Rural	Kwazulu Natal
Van Wyk (2004) [11]	1999	1995-1999	4-5	599	1219			Urban/Rural	Gauteng
Van Wyk (2004) [11]	1999	1995-1999	6	284	476			Urban/Rural	Gauteng
Van Wyk (2004) [11]	1999	1995-1999	4-5	418	1019			Urban/Rural	North West
Van Wyk (2004) [11]	1999	1995-1999	6	553	1058			Urban/Rural	North West
Van Wyk (2004) [11]	1999	1995-1999	4-5	285	708			Urban/Rural	Mpumalanga

Van Wyk (2004) [11]	1999	1995-1999	6	392	698			Urban/Rural	Mpumalanga
Van Wyk (2004) [11]	1999	1995-1999	4-5	273	679			Urban/Rural	Limpopo
Van Wyk (2004) [11]	1999	1995-1999	6	423	753			Urban/Rural	Limpopo
Wanjau (2006) [52]	2006	2005-2009	3	15	59			Rural	Mpumalanga
Wanjau (2006) [52]	2006	2005-2009	4	43	77			Rural	Mpumalanga
Wanjau (2006) [52]	2006	2005-2009	5	71	133			Rural	Mpumalanga
Williams (1985) [53]	1981	1980-1984	2	29	101	0.9	2	Urban	Gauteng
Williams (1985) [53]	1983	1980-1984	2	25	99	0.7	1.5	Urban	Gauteng
Williams (1985) [53]	1981	1980-1984	3	125	241	1.8	2.7	Urban	Gauteng
Williams (1985) [53]	1983	1980-1984	3	83	207	1.5	2.6	Urban	Gauteng
Williams (1985) [53]	1981	1980-1984	4	239	329	2.3	3.2	Urban	Gauteng
Williams (1985) [53]	1983	1980-1984	4	167	284	2.4	3.3	Urban	Gauteng
Williams (1985) [53]	1981	1980-1984	5	238	329	3.9	4	Urban	Gauteng
Williams (1985) [53]	1983	1980-1984	5	167	284	2.8	3.6	Urban	Gauteng
